# The Comparison of Clinical Result between Primary Repair of the Anterior Cruciate Ligament with Additional Internal Bracing and Anatomic Single Bundle Reconstruction—A Retrospective Study

**DOI:** 10.3390/jcm10173948

**Published:** 2021-08-31

**Authors:** Dawid Szwedowski, Łukasz Paczesny, Jan Zabrzyński, Maciej Gagat, Marcin Domżalski, Gazi Huri, Wojciech Widuchowski

**Affiliations:** 1 Orthopaedic Arthroscopic Surgery International (OASI) Bioresearch Foundation, 20133 Milan, Italy; 2 Citomed Healthcare Center, Department of Orthopaedics, Orvit Clinic, Sklodowskiej 73, 87-100 Toruń, Poland; drpaczesny@gmail.com (Ł.P.); zabrzynski@gmail.com (J.Z.); 3 Department of General Orthopedics, Musculoskeletal Oncology and Trauma Surgery, University of Medical Sciences, 61-701 Poznan, Poland; 4 Department of Histology and Embryology, Faculty of Medicine, Collegium Medicum in Bydgoszcz, Nicolaus Copernicus University in Torun, 85-067 Bydgoszcz, Poland; mgagat@cm.umk.pl; 5 Department of Orthopedic and Traumatology, Veterans Memorial Hospital, Medical University of Lodz, 90-549 Lodz, Poland; marcindomzalski@yahoo.com; 6 Orthopaedics and Traumatology Department, Hacettepe Universitesi, Ankara 06100, Turkey; gazihuri@yahoo.com; 7 Department of the Knee Surgery, Arthroscopy and Sports Traumatology, District Hospital of Orthopedics and Trauma Surgery, 41-940 Piekary Slaskie, Poland; sportmed@sportmed.com.pl

**Keywords:** anterior cruciate ligament (ACL), primary ACL repair, internal bracing, knee laxity

## Abstract

Background: The current standard of treatment of anterior cruciate ligament (ACL) is reconstruction (ACLR). This technique has some disadvantages: poor proprioception, donor site morbidity and the inability to restore joint kinematics. ACL repair could be an alternative treatment. The purpose of the study was to compare the stability and the function after ACL primary repair versus single-bundle ACLR. Methods: In a retrospective study, 12 patients underwent primary ACL repair with internal bracing, 15 patients underwent standard ACLR. Follow-up examinations were evaluated at up to 2 years postoperatively. One patient in the ACL repair group was lost to follow-up due to re-rupture. The absolute value of anterior tibial translation (ATT) and the side-to-side difference in the same patient (ΔATT) were evaluated using the GNRB arthrometer. The Lysholm knee scoring was obtained. Re-ruptures and other complications were recorded. Results: Anterior tibial translation (ATT) was significantly decreased in the ACL repair group compared with the ACLR group (5.31 mm vs. 7.18 mm, respectively; *p* = 0.0137). Arthrometric measurements demonstrated a mean side-to-side difference (ΔATT) 1.87 (range 0.2 to 4.9) mm significantly decreased compared to ACLR 3.36 (range 1.2–5.6 mm; *p* = 0.0107). The mean Lysholm score was 85.3 points in the ACL repair group and 89.9 in ACLR group. No significant differences between ACL repair and ACLR were found for the Lysholm score. There was no association between AP laxity and clinical outcomes. There were two complications in the internal bracing group: one patient had re-rupture and was treated by ACLR, another had limited extension and had arthroscopic debridement. Conclusions: Anterior tibial translation was significantly decreased after ACL repair. Additionally, the functional results after ACL repair with internal bracing were comparable with those after ACLR. It should be noted that the two complications occurred. The current study supports further development of ACL repair techniques.

## 1. Introduction

Responsible for controlling anterior–posterior (AP) and rotatory knee laxity, the anterior cruciate ligament (ACL) is the major knee joint stabilizer. The growing popularity of recreational and competitive sport activities has contributed to an increased number of ACL injuries in the past three decades. Due to the limited healing potential of the ligament, ACL reconstruction (ACLR) is the gold standard in ACL injury treatment [1]. The administration of platelet-rich plasma (PRP), which comprises growth factors and displays evident biological activity [2,3], showed promise in stimulating ACL healing. However, PRP augmentation has failed to provide superior functional results in combination with ACLR [4,5]. Thus, improved patient selection and the latest advances in arthroscopic techniques, such as internal bracing, have aroused renewed interest in ACL repair [6,7,8,9]. These methods offer potential advantages, including native ACL preservation, reduced proprioception loss and no autologous graft harvesting-associated morbidity.

Furthermore, some authors contend that there may be a lower risk of posttraumatic osteoarthritis after repair [10,11]. The rationale for internal bracing augmentation is to diminish the gap between the injured sites and protect ACL healing during early mobilization and rehabilitation [7,12,13]. Additionally, it has been demonstrated in preclinical studies that providing repaired tissue with some mechanical support resulted in the enhancement of the biomechanical properties of the ACL [14,15,16]. Despite the fact that researchers have not reported any exact threshold for anterior knee laxity that would allow for scar tissue attachment back to the bone, increased anteroposterior translation can result in compromised healing capability and an inability to form stable scar tissue [17,18,19]. The purpose of this study was to evaluate the clinical results after ACL repair compared to single-bundle ACLR. It was hypothesized that arthroscopic ACL repair with additional internal bracing would result in good knee stability and subjective outcomes comparable to ACL reconstruction. Considering that one of the major problems during ACL repair is insufficient postoperative knee stability, we decided to focus on this particular issue.

## 2. Materials and Methods

**Figure 1:**
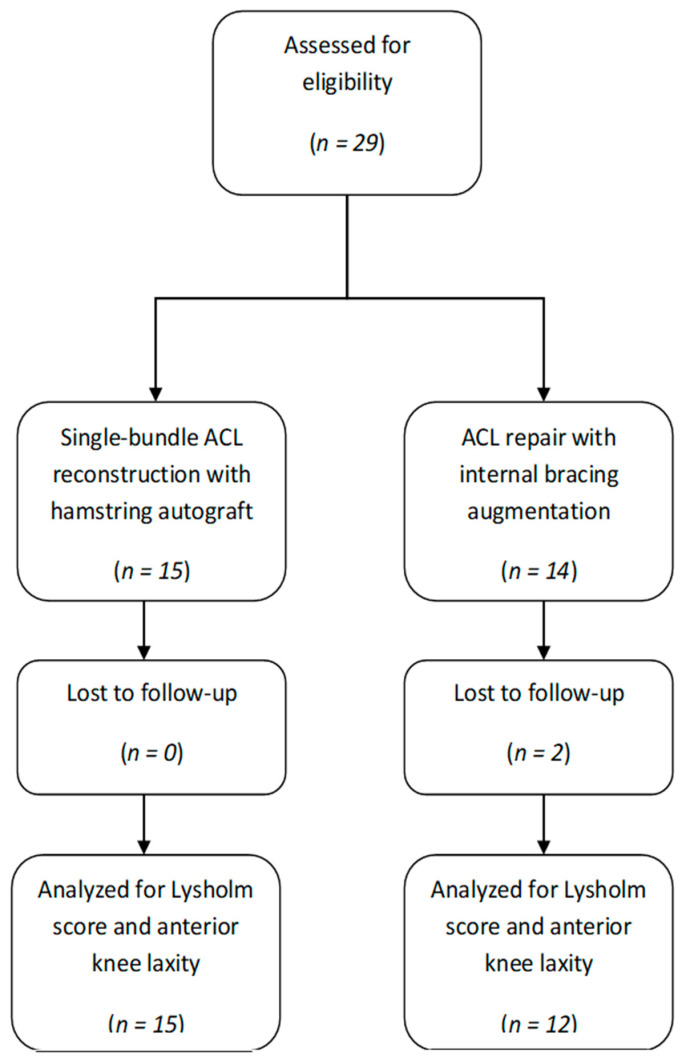
The flowchart presenting the study design.

We retrospectively followed a group of 12 patients who underwent primary ligament repair with internal bracing augmentation and 15 patients who underwent single-bundle ACL reconstruction to have complete ACL tears treated at our institution between 2017 and 2019 (Figure 1).

The inclusion criteria consisted of an acute, complete ACL injury, diagnosed within 6weeks of injury, in patients with symptomatic instability. The exclusion criteria consisted of ACL tears in which the ligamentous fibers could not be reapproximated with suture-bracing, ≤G1 pivot-shift instability, significant malalignment, chondral defect or meniscal injury, multiligament injury, contralateral knee injury. All patients with ACL ruptures were assessed with MRI. The possibility of internal bracing repair was discussed with the patient in the event that femoral side tear was found. If the patient revealed interest in this kind of treatment, the final decision was made depending on the exact quality and mobility of the stump.

Despite a lack of patient randomization, parameters such as age, sex and time since injury were similar in both the study group, which underwent ACL repair and the control group, which underwent ACLR.

The senior author (Ł.P.) performed all procedures conducted within 8 weeks of the injury. Non-operative and operative treatment protocols were discussed with each patient prior to treatment. Standard ACL reconstruction methods were specifically discussed as an alternative to primary repair with internal bracing. Patients who did not consent to the ACL repair method underwent standard ACL reconstruction with hamstring autografts. The Lysholm Knee Questionnaire was used to conduct a retrospective follow-up of all patients. Anterior knee laxity was evaluated postoperatively by an independent examiner who measured the side-to-side difference in anterior tibial translation in millimeters with a GNRB arthrometer (Genourob, Laval, France). Throughout the follow-up, postoperative complications, recurrent instability and reinjury were documented. The study protocol was approved by the local institutional ethics committee.

### 2.1. Surgical Technique

**Figure 2:**
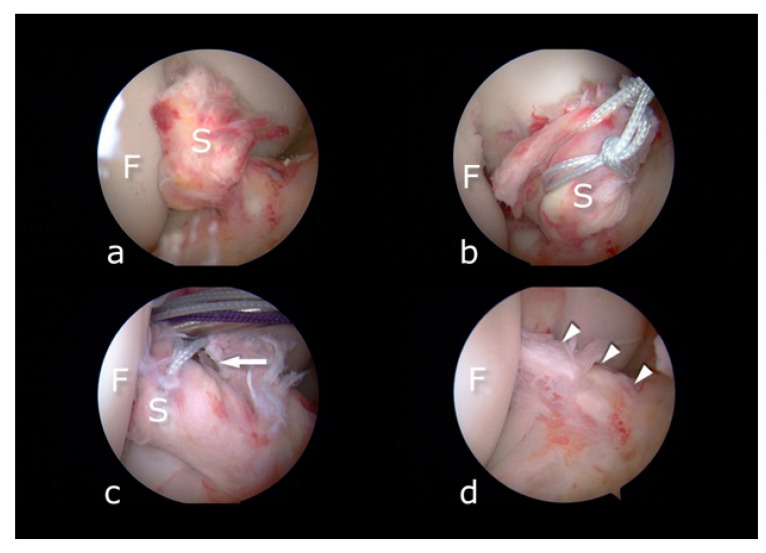
(**a**) Fresh ACL rupture. Stump (S) is waving in front of the intercondylar notch of the femur (F). (**b**) The stump (S) is sutured by means of the closed-loop FiberLink sutures and retracted from the femoral bone (F). (**c**) After preparation of the femoral tunnel the stump (S) is repositioned to meet native insertion on the femur (F). Next, the tibial tunnel is drilled. The drill tip is visible in the center of the stump (arrow). (**d**) After completion of the procedure, the tension of the repaired ACL (arrowheads) and FiberTape is checked and a full range of motion confirmed.

Patients were positioned supine, as is typical of knee arthroscopy and standard ACL reconstruction and were administered a spinal or general anesthetic. Under anesthesia, the patient’s knee was examined to establish whether its instability corresponded with ACL insufficiency. Diagnostic arthroscopy was performed to examine the ACL and to confirm a complete tear amenable to the primary repair technique. The Lachman test was performed under direct visualization. Patients with a complete ACL tear that had sufficient distal remnant length and tissue quality to reapproximate the remnants underwent ACL repair. ACL repair with the internal bracing technique has been described in previously published technical notes [20]. Primary ligament repair is depicted in Figure 2.

The standard ACL reconstruction technique was performed in line with Siebold’s guidelines [21] in the same patient setting after arthroscopic evaluation of the joint. After complete ACL injury was confirmed, a 3 cm oblique incision over the hamstring insertion was made and the semitendinosus plus gracilis tendons were harvested. The graft was double folded, prepared and measured and the femoral tunnel was drilled in compliance with the anteromedial technique aiming at femoral insertion of the native ACL. Next, the tibial tunnel was drilled with the use of an elbow guide. The graft was passed through and anchored on the femoral cortex using the Endobutton system (*Smith and Nephew, USA*). Distal fixation was achieved with a titanium RCI screw whose diameter corresponded with that of the graft.

### 2.2. Rehabilitation Protocol

Although external braces were not used in the internal bracing group, the ACL reconstruction group was provided with external hinge braces for 6 weeks without limiting the range of motion (ROM) (Protect 4 Evo, Medi, Bayreuth, Germany). The same rehabilitation protocol described by Królikowska et al. [22,23,24] was used in both groups. Partial weight-bearing with the assistance of crutches was allowed as tolerated. Progression to weight-bearing was permitted after 3 weeks, while the full active range of motion was allowed 6 weeks after the surgery.

### 2.3. Anterior Knee Laxity Measurement

Several arthrometers are widely used to measure knee joint sagittal stability; the KT-1000 (MED metric, San Diego, CA, USA) and the Rolimeter (Aircast, Summit, NJ, USA) are the most popular devices at present because of their simplicity of use. The KT-1000 and the Rolimeter [25,26] are equally reliable and operator-dependent [27,28,29,30]. Considerably more reproducible than the other tools, regardless of the tester’s experience [31,32], the GNRB arthrometer was used to objectively assess anterior tibial translation. As an additional benefit, the measurement was achieved independent of the uninvolved side [32].

**Figure 3:**
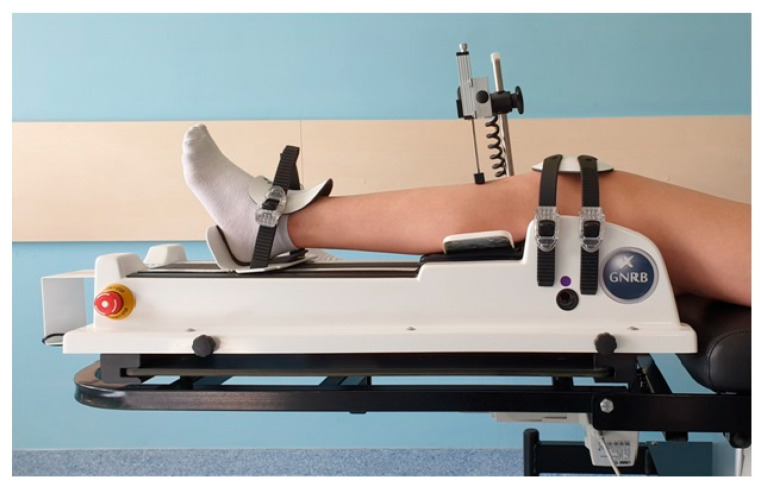
The patient was lying on a standard examination table in the supine position with the arms placed along the body, each knee being comparatively tested, the healthy knees are investigated first. The lower limb is placed in rigid adjustable leg support, with the knee placed at 0° of rotation. The knee should be placed so that the inferior pole of the patella corresponds to the lower border of the patellar support, the joint line is palpated and should be located between the support and the jack.

Reproducing the Lachman test position (Figure 3), the GNRB is a knee laxity testing device that measures AP tibial translation at 20° of knee flexion.

The AP laxity measurement was performed using a protocol previously described by Nouveau et al. [33].

### 2.4. Statistical Analysis

Data analysis was performed with MedCalc Statistical Software (version 16.4.3; MedCalc Software, Ostend, Belgium), while Shapiro–Wilk tests were used to assess the normality of continuous variables. The correlation of variables within patients was established with the Spearman Rho correlation coefficient and the Mann–Whitney U test served to enable the statistical comparison of independent data. All comparisons between groups and the statistical analysis were blinded and performed by an independent investigator. A *p*-value < 0.05 was considered statistically significant. The sample size for our study was based on the results of a previous study on ACL primary repair [33]. A sample size calculation showed that 12 patients per group would be required to detect a difference in anterior knee laxity with a standard deviation of two points with a power of 80% and a significance level of α = 0.05.

## 3. Results

**Figure 4:**
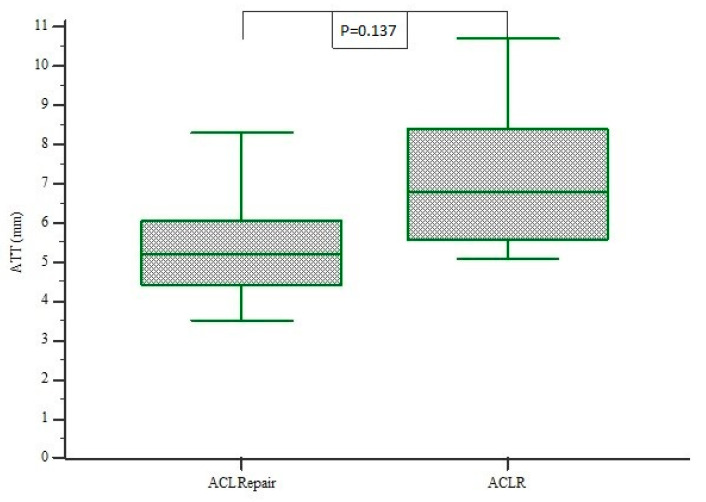
Box plot of assessments of anterior tibial translation (ATT) comparing the difference between ACL repair and ACLR group. ATT was significantly decreased in the ACL repair group compared with the ACLR group (5.31 mm vs. 7.18 mm, respectively; *p* = 0.0137).

In the examined cohort, 12 patients underwent internal bracing repair of the ACL, while 15 patients underwent ACL reconstruction. One patient in the ACL repair group was lost to follow-up due to re-rupture. The mean age in the ACL repair group was 36 (range: 15–55) years. This study group included six males and five females (seven right knees and four left knees). All of the patients suffered from an acute knee injury 1–2 months preoperatively. The average time from surgery to follow-up in the ACL repair with additional internal bracing group was 14.8 (range 5–24) months and for patients treated with ACLR, 13.6 (range 10–24) months. There were no statistically significant differences of the demographic data between two groups. Anterior tibial translation (ATT) was significantly decreased in the ACL repair group compared with the ACLR group (5.31 mm vs. 7.18 mm; *p* = 0.0137) (Figure 4).

**Figure 5:**
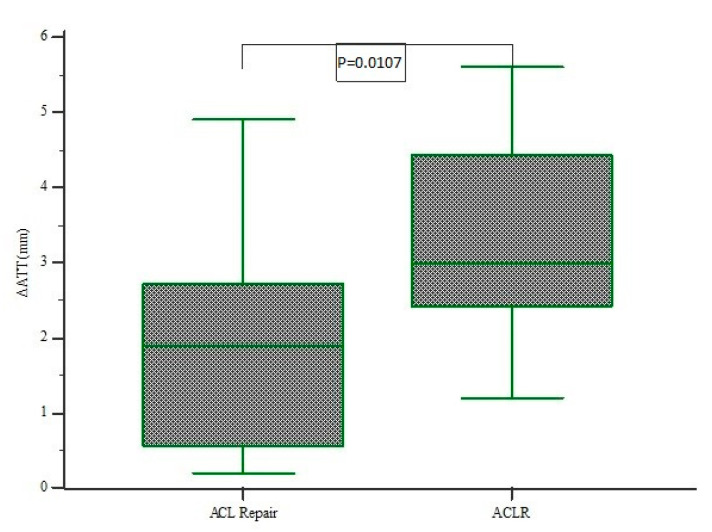
Box plot of assessments of side-to-side difference (ΔATT) in ACL repair and ACLR group. It demonstrates a mean side-to-side difference 1.87 (range 0.2 to 4.9) mm in ACL repair significantly decreased compared to ACLR 3.36 (range 1.2 to 5.6 mm; *p* = 0.0107).

Moreover, GNRB measurements demonstrated a significantly decreased mean side-to-side difference (ΔATT) at 1.87 (range 0.2 to 4.9) mm when compared to ACLR at 3.36 (range 1.2 to 5.6 mm; *p* = 0.0107) (Figure 5).

**Figure 6:**
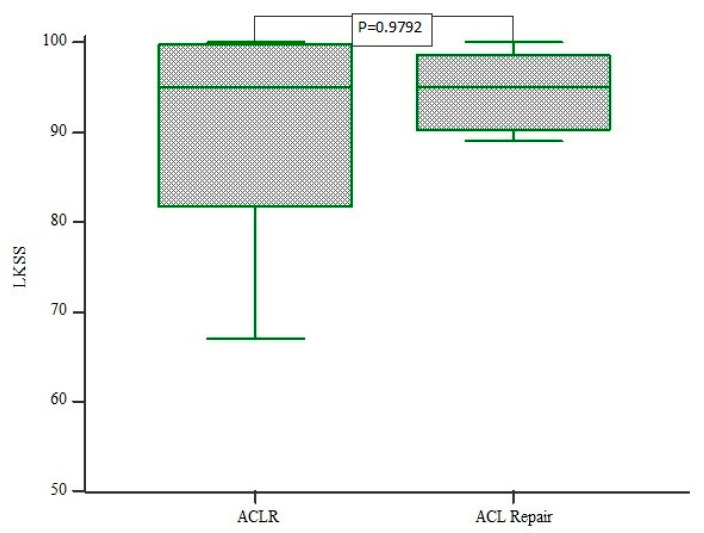
Box plot of Lysholm score assessments comparing ACL repair and ACLR group. The mean Lysholm score after 12 months was 89.2 (range: 57–100) in the internal bracing cohort and 89.9 (range: 67–100) in the ACLR group. There were no statistically significant differences between both groups (*p* = 0.9793).

The mean Lysholm score after 12 months was 89.2 (range: 57–100) in the internal bracing cohort and 89.9 (range: 67–100) in the ACLR group. No significant differences between ACL repair and ACLR were found for the Lysholm score (*p* = 0.9793) (Figure 6). Additionally, no correlation was demonstrated between Lysholm scores and ATT for either surgical technique (*p* > 0.05).

**Figure 7:**
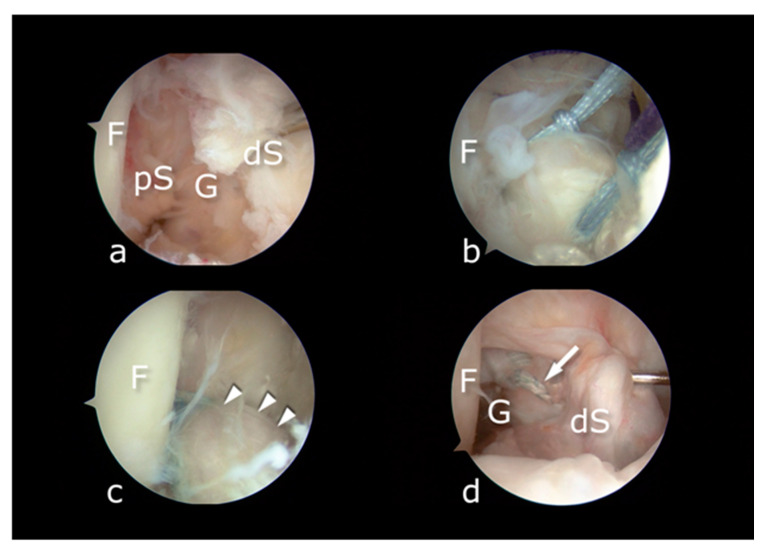
(**a**) Primary ACL repair. Distal stump (dS) is waving in front of the intercondylar notch of the femur (F). Proximal stump (pS) is visible. (**b**) The stump is sutured by two closed-loop FiberLink sutures and retracted from the femoral bone (F). (**c**) After completion of the procedure, the tension of the repaired ACL (arrowheads) and FiberTape is checked. (**d**) Revision surgery. Re-rupture of the construct is visible with gap (G) opening. Distal stump is folding and hook traction reveals FiberLink sutures (arrow).

Two complications occurred in the internal bracing cohort: one patient suffered from re-rupture 8 months after the primary surgery and was treated with ACLR (Figure 7), while the other had limited extension and underwent arthroscopic debridement of scar tissue in the intercondylar notch.

**Figure 8:**
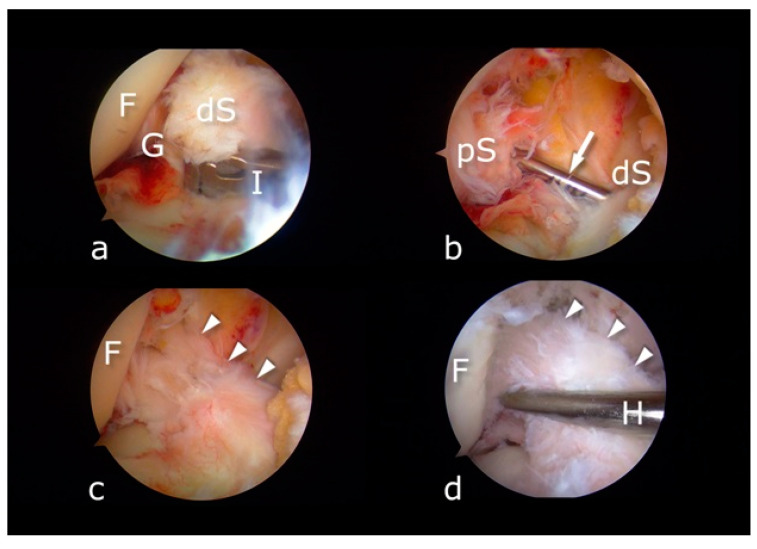
(**a**) Primary ACL repair. Distal stump (dS) waving in front of the intercondylar notch of the femur (F) with a gap (G) opening after retraction by suturing instrument (I). (**b**) Proximal stump (pS) drilled (arrow). Distal stump (dS) was retracted at this stage. (**c**) After completion of the procedure, the tension of the repaired ACL (arrowheads) and FiberTape was confirmed. (**d**) Second look surgery due to poor range of motion. After scaring tissue removal physiological tension of the healed ACL was confirmed by hook (H) traction.

Second-look arthroscopy in the latter case revealed a well-healed ACL (Figure 8). There were no wound infections during the follow-up period.

## 4. Discussion

The most essential finding in the study was that ACL repair with internal bracing resulted in significantly smaller anterior knee laxity than ACLR. In a systemic review, Kandhari et al. emphasized that the parameters of knee laxity were not explicitly reported in previous clinical ACL repair studies [34]. AP laxity is important because it is associated with the extent of tibiofemoral cartilage damage after ACL surgery [35]. Several studies have demonstrated good knee stability measured on the KT-1000 [36,37,38,39,40,41,42,43] and the Rolimeter [44] after primary ACL repair. Although a majority of clinical studies use these tools for anterior tibial translation, it must be kept in mind that these devices are examiner dependent. Collete et al. demonstrated that GNRB revealed superior intra- and inter-examiner reproducibility over KT-1000. Additional advantages of this tool include better control of the magnitude, direction and rate of force application as well as hamstring activity [32]. Other studies demonstrated that ACL tears were diagnosed more accurately with the GNRB than with the Telos stress device (Gmbh, Hungen/Obbornhafen, Germany) [13,31]. However, in a more recent study, no significant differences were found between these two instruments in reproducibility for measuring anterior tibial translation without rotation in normal knees [45]. Comparing the GNRB with the computer navigation system, Jenny et al. validated the device and concluded that the differences between the measurements made with the use of the GNRB and the navigation system were insignificant and unlikely to have any clinical impact [42]. To the best of our knowledge, this is the first study using a GNRB arthrometer to measure anterior tibial translation after ACL repair.

The loss of ACL stability poses a major challenge to ACL repair efficacy. Out of several different techniques proposed, such as sutures [43] and anchors [36,37,38,42], augmenting the primary repair construct with internal sutures provides additional stability and better healing potential [14,15,16]. Murray et al. showed that providing additional stabilization between the tibia and the femur contributes to the enhancement of the structural properties of ACL repairs when compared with the suture technique [14]. Similarly, Seitz et al. demonstrated superior biomechanical properties, such as stiffness and tensile strength and less AP laxity for ACL repair with polyethylene terephthalate band augmentation [15]. On the basis of a porcine model, Fleming et al. [13] showed that isolated augmentation resulted in knee laxity values that were within 0.5 mm of the intact ACL joint when the sutures were tied with the knee at 60° flexion. Laxity values were evaluated after cyclical shear loading in compliance with the Lachman test and primary suture repair without augmentation significantly increased anterior laxity compared with the normal knee. In a recent study, Bachmaier et al. [19] proved the internal brace to be a protective primary stabilizer and provided reduced peak loads on ACL repair as well as gap formation of less than 3 mm because it possessed a synergistic load-sharing function with ACL repair in a porcine model. By the simulation of loads up to 350 N experienced during late rehabilitation, they concluded that the internal bracing of ACL repair ensured adequate stabilization to protect the healing ACL at loads affecting an intact knee during usual everyday activity. Another cadaveric study showed that anterior knee laxity after augmented ACL repair was comparable to the range of an intact knee [38].

Furthermore, we did not detect any disparity in clinical outcomes between ACL repair and ACLR. Recent studies have demonstrated that primary repair may lead to good clinical results. For example, Van Heusden et al. reported excellent outcomes of primary repair with additional internal bracing in 42 patients with 4.8% failures at a 2-year follow-up [13], while Jonkergouw et al. used internal bracing augmentation in some patients (27/56) who underwent a suture anchor repair technique for proximal ACL tears. No significant discrepancy in clinical outcome scores or failure rates at a mean follow-up of 38.4 months was demonstrated [8]. Similarly, our study did not show any major difference between ACLR and ACL repair in clinical outcomes.

Both complications that occurred in the ACL repair group, i.e., re-ruptures and a limited range of motion can be connected with the over stiffened construct of the repaired ACL. With high success rates for return-to-play typically reported [45], ACLR is the procedure of choice in ACL insufficiency treatment; nevertheless, manifest disadvantages of this procedure comprise a loss of proprioception, donor site morbidity, incomplete return to high-demand sports and the inability to restore normal kinematics of the knee joint [42,43]. In view of the fact that ACL injuries typically occur in a younger demographic, new approaches that can restore the near-anatomic function of this ligament have the potential to become widely used in clinical practice.

Despite several strengths, the major limitations of our study are the small number of patients included, short follow-up duration and retrospective analysis of data. Another limitation is the lack of MRI and 2nd look arthroscopic findings in all cases. Additionally, the fact that the GNRB arthrometer is a robotic device and is thus very sensitive to changes in positioning could lead to low interrater reliability of this tool. It should also be noted that patients were not randomized; therefore, further studies are required.

## 5. Conclusions

The clinical results of ACL repair are similar to those after ACL reconstruction, which was also confirmed by other comparative studies. Although we did not detect any major disparities between ACL repair with internal bracing and anatomic single-bundle reconstruction with respect to objective knee laxity, we noticed some complications in the ACL repair group.

## Data Availability

The data from this study are available from the corresponding author upon reasonable request.
